# Robotic Retinal Surgery Impacts on Scleral Forces: In Vivo Study

**DOI:** 10.1167/tvst.9.10.2

**Published:** 2020-09-01

**Authors:** Müller G. Urias, Niravkumar Patel, Ali Ebrahimi, Iulian Iordachita, Peter L. Gehlbach

**Affiliations:** 1 Wilmer Eye Institute, Johns Hopkins Hospital, Baltimore, MD, USA; 2 Federal University of Sao Paulo, São Paulo, Brazil; 3 Laboratory for Computational Sensing and Robotics, Johns Hopkins University, Baltimore, MD; 4 Whiting School of Engineering, Johns Hopkins University, Baltimore, MD, USA

**Keywords:** robotic surgical procedures, microsurgery, retina

## Abstract

**Purpose:**

This study aims to map force interaction between instrument and sclera of in vivo rabbits during retinal procedures, and verify if a robotic active force control could prevent unwanted increase of forces on the sclera.

**Methods:**

Experiments consisted in the performance of intraocular movements of a force sensing instrument, adjacent to the retinal surface, in radial directions, from the center to the periphery and back, and compared manual manipulations with robotic assistance and also robotic assistance with an active force control. This protocol was approved by the Animal Use and Ethical Committee and experiments were according to ARVO Statement of Animal Use.

**Results:**

Mean forces using manual manipulations were 115 ± 51 mN. Using robotic assistance, mean forces were 118 ± 49 mN. Using an active force control method, overall mean forces reduced to 69 ± 15, with a statistical difference compared with other methods (*P* < 0.001). Comparing intraocular directions, superior sector required higher forces and the force control method reduced differences in forces between users and retained the same force pattern between them.

**Conclusions:**

Results validate that the introduction of robotic assistance might increase the dynamic interactions between instrument and sclera, and the addition of an active force control method reduces the forces at levels lower than manual manipulations.

**Translational Relevance:**

All marketing benefits from extreme accuracy and stability from robots, however, redundancy of safety mechanisms during intraocular manipulations, especially on force control and surgical awareness, would allow all utility of robotic assistance in ophthalmology.

## Introduction

Robotic devices for ophthalmic purposes raised attention, as those systems may improve capabilities in the constrained, micrometric, and unforgiving intraocular space. Since 1989, when the first device for ocular procedures was published,^1^ technology improved and motivation increased, leading to the first clinical robotic platform being tested on humans and published in 2018.^2^^,^^3^ However, increased concerns have raised due to the possible impact of robotic devices on force feedback and instrument awareness.^4^^–^^7^ Therefore, along the exciting marketing of human studies and the possible future upcoming, there are requirements to understand how the user, instrument, sclera, and – now- the robot interact with each other and modifies the surgical awareness.

During retinal procedures, surgeons often are required to perform coordinated instrument movement, and most of that coordination relies on instrument-sclera forces feedback - described in the order of milli-Newtons.^8^^–^^10^ Human force sensing capabilities on ophthalmic procedures are described to be at its low boundaries at 7.5 mN, the same amount of force described to generate a tear in rabbits’ eyes.^11^ Other factors might also impact on surgical awareness, for example, surgical field of view, instrument shadow, and surgeons “mechanical memory” after years of training.^12^^–^^15^ Therefore, an impact on sensing capabilities with the robotic introduction may compromise surgical and instrument awareness, which might lead to unwanted outcomes.

A cooperative robotic system, for example, in which the surgeon directly manipulates the instrument at the robot end-effector, uses the rigid robot arm and a force/torque sensor to filter out small force transmissions – in benefit of stability, accuracy, and tremor removal.^16^ A different system, called master-slave (or tele-operated), uses a joystick to remotely control an instrument, and any force feedback would be filtered out as well, but with the additional capability to scale movements.^17^ On both, instrument mechanisms and robotic arm are supposed to be steady, and any unexpected movement, either from the patient's head and/or the eyeball, could generate undesirable interactions that the robot -without additional systems – cannot react as a free-hand human user would.

Previous studies using dry phantoms models and ex vivo eyes shows that, besides the logical diminishing of the force feeling, there are increased forces between instrument and sclera with the introduction of the robot.^18^^–^^24^ However, there are no in vivo studies that present how instrument and sclera interact during intraocular instrument movement with robotic assistance. The objectives of this study are to map forces between the sclera and instrument tool-shaft using force sensing instruments, to verify changes in force pattern using robotic assistance, and test how a robotic force control could address those interactions. Despite a growing market and numerous technology possibilities, this force assessment might be crucial to verify the relevance of force interaction as a factor in robotic implementation and to emphasize the importance of instrument surgical awareness toward a progressive safety use of robotic assistance.

## Methods

This research was performed at the Johns Hopkins University (Wilmer Eye Institute and Laboratory of Computational Sensing and Robotics) and was supported by the National Institutes of Health under grant 1R01EB023943-01. This project adopted New Zealand rabbits as the animal model due to their extraocular muscle's similarities with humans, easy handling, and possibility to increase sample size. The protocol was approved by the Animal Use and Ethical Committee from Johns Hopkins University and experiments were according to Association for Research in Vision and Ophthalmology Statement of Animal Use. The study used a force sensing instrument and a cooperative robotic platform to perform radial and circumferential intraocular movements, adjacent to the retinal surface. The components of the setup and tasks are described below.

### Experimental Setup

#### Force Sensing Instrument

To measure force values, authors implemented a multipurpose sensing instruments consisting of a 3D printed handgrip, a metal tool shaft with optical fibers, and a stainless-steel cannula at the instrument tip. Three FBG optical fibers are distributed at 120-degree intervals around the tool shaft with capabilities to quantify tool-shaft and tool-tip forces separately. FBG technology works through changes in reflection of a grating - a modulation generated by broadband light - transmitted inside of a photosensitive optical fiber.^25^ Changes in strain, temperature, acceleration, pressure, and displacement, generates a wavelength shift on those transmitted reflections, and then – if other variables are considered fixed - are correlated to force. Advantages of FBG include a reduced fiber diameter (between 60 and 200 µm), high sensitivity, sub-millinewtons resolution, besides electrically isolation and electromagnetic immunity.^25^ Some of their disadvantages include temperature influence and reduced resolution for axial interactions. The tool was debiased from acceleration, temperature, pressure, and movement before all movements. Forces applied to the optical fibers generate variations in the wavelength, were captured by an interrogator (si155; Micron Optics Inc., Atlanta, GA), and sent to the computer, available to robotic decisions.

#### Robotic Platform

**Figure 1.**
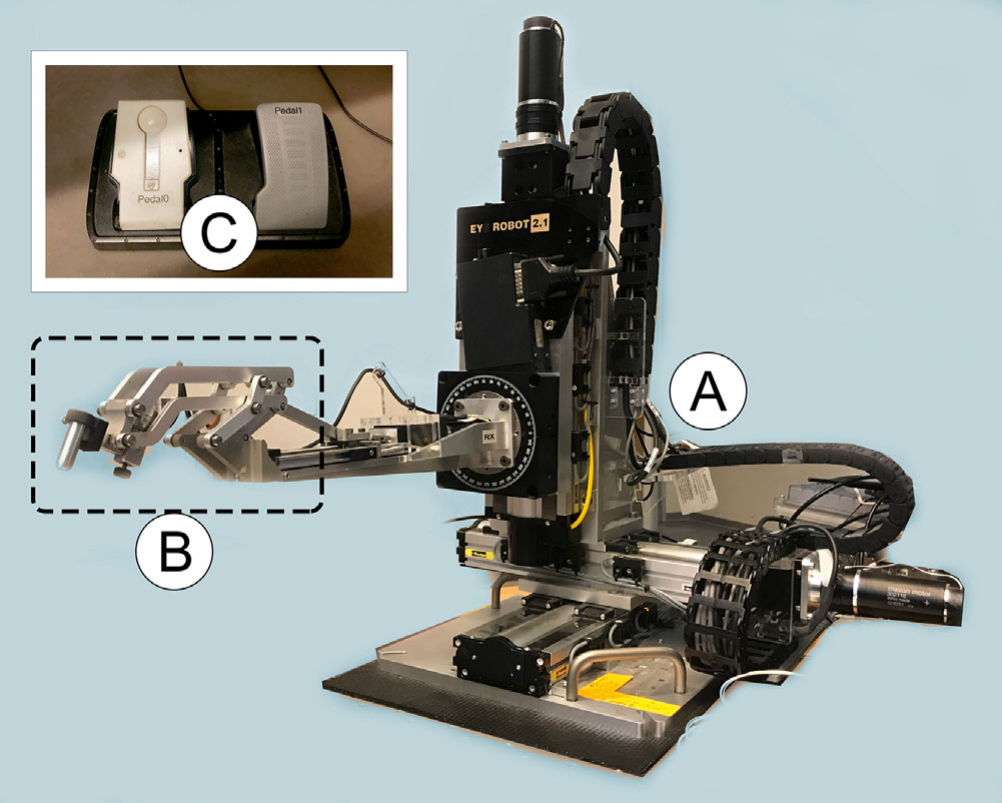
(**A**) Steady hand eye robot (SHER), a 5-degrees-of-freedom robotic platform. (**B**) Robot end-effector, with the tilting mechanism and slot for instrument insertion. (**C**) Pedal for admittance control.

The robotic platform adopted in this study was the Steady Hand Eye Robot 2.1, developed at the Johns Hopkins University. This is a cooperatively controlled system with 5-Degrees-of-Freedom (DOF), designed for retinal procedures.^25^ This system relies on a foot-pedal switch to adjust the admittance of the movement; in other words, as the pedal is pushed, it progressively removes restrictions on the end-effector movement and progressively sets the instrument free for the desirable movement. As expected, if the pedal is not pressed, the instrument is locked in position. The robot and foot pedal are illustrated in Figures 1^2^A and 1C, respectively. At any time, the surgeon can detach the instrument from the end effector handle (Fig. 1B) or press an emergency button to stop the robot from acting. This system records not only handle position and movement velocities, but also processes force feed from interrogators and uses that data if wanted by the user to react accordingly.

**Figure 2.**
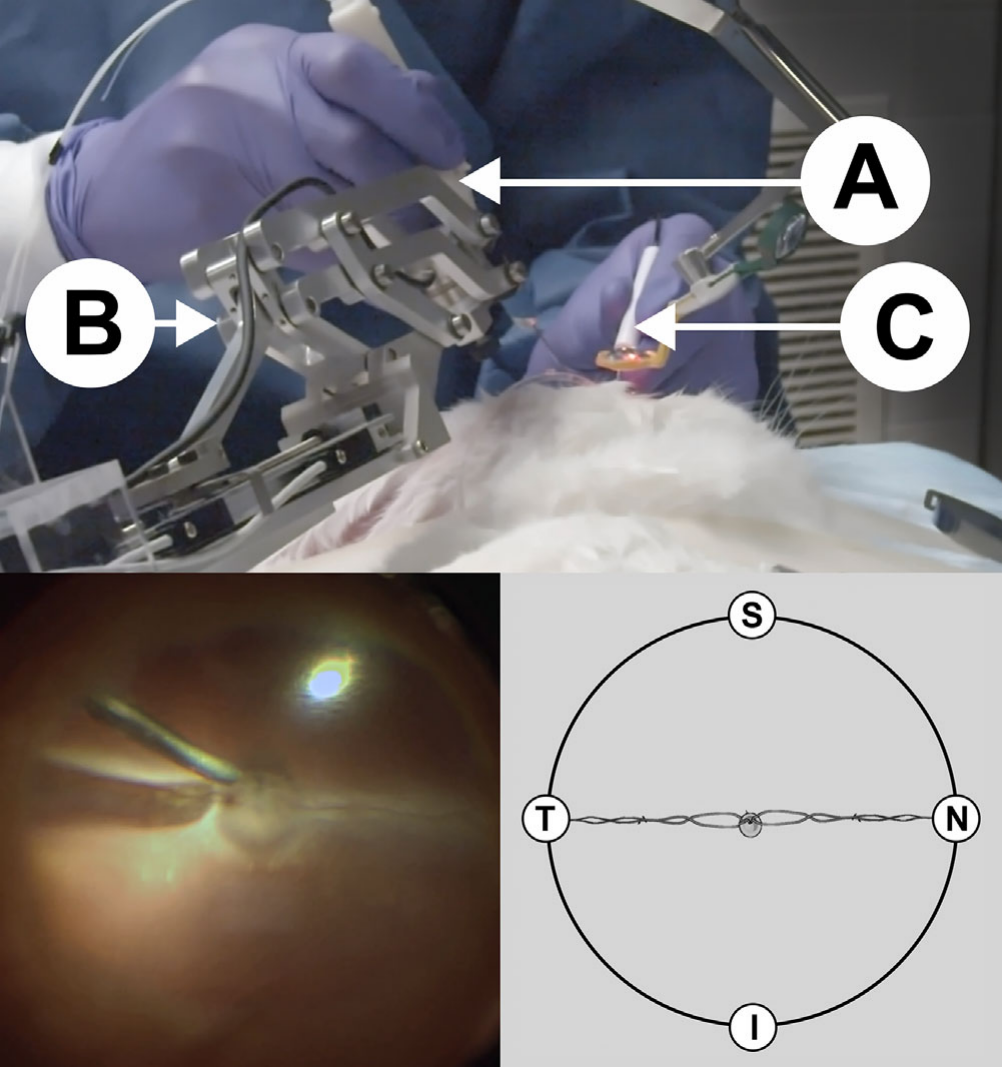
*Top*: Procedure using robotic assistance (**A**) force sensing instrument; (**B**) robot end-effector; (**C**) light pipe). *Bottom left*: Rabbit fundus viewing during intraocular movements. *Bottom right*: Schematic viewing of directions according to the optic disk (*center*) and vessels; S = superior (close to the user); T = temporal (close to the robot); I = inferior (away from the user); N = nasal (away from the robot).

#### In Vivo Setup

A sample size of 14 New Zealand white of rabbits (approximately 3.5–4.0 kg) were used, with a total of 28 eyes available for this research. Mydriatic eyedrops (Cyclopentolate 2% and Phenylephrine 10%) were used 30 minutes previous to anesthesia. Then, rabbits were anaesthetized with Xylazine 20 mg/mL and Ketamine 100 mg/mL intramuscularly. Eyelid canthotomy was performed to properly expose the eyeball. Infusion trocar was set temporally, using 27-gauge trocars. Auxiliary sclerotomy was done and trocar placed. With a distance of 120 degrees from auxiliary trocar, the main sclerotomy was done using a 20-gauge vitrectomy blade, in order to further insert the 20-gauge force sensing instrument. Surgeon performed pars plana phacofragmentation, and then pars plana vitrectomy (PPV) using EVA Vitrectomy System (D.O.R.C. Dutch Ophthalmic Research Center International B.V.), with the intraocular pressure set to 20 mm Hg. After PPV, the surgeon inserts the instrument and tasks were started.

### Experiments

#### Tasks

Intraocular tasks were performed by two retina specialists (the first had 2 years of retina surgery experience and the second had +20 years of retina surgery experience) and consisted of intraocular movements with the instrument tip adjacent to the retinal surface, in randomized radial directions (inferior, temporal, superior and nasal) from the optic disc (reference) to the ora serrata and back (Fig. 2), with a total of 120 movements per eye.

#### Measures

Force information was with regard to the scleral distortion – strain – at scleral port and was collected every 5 ms (200 Hz) by the aforementioned interrogators. Authors developed a force control method using feed from sensors applied to robot's kinematics to control unwanted increase of forces by the robot. Previously described and tested on dry phantoms, we now validate results in this in vivo experiment. Therefore, this paper compares forces during intraocular tasks between manual, robot-assisted manipulation with no force control (RA-NFC), and robot-assisted manipulation with force control (RA-FC).

### Analysis

All logfiles from sensors were compiled after experiments using Tableau Desktop 2019 (Tableau Software, Seattle, WA), and image data from microscope was also used to confirm movements in the post-analysis. Authors performed statistical analysis using IBM SPSS Statistics (IBM Corporation, Armonk, NY), using ANOVA to compare three groups. Chi-squared analysis was performed to verify the relationship between nominal variables.

## Results

**Figure 3.**
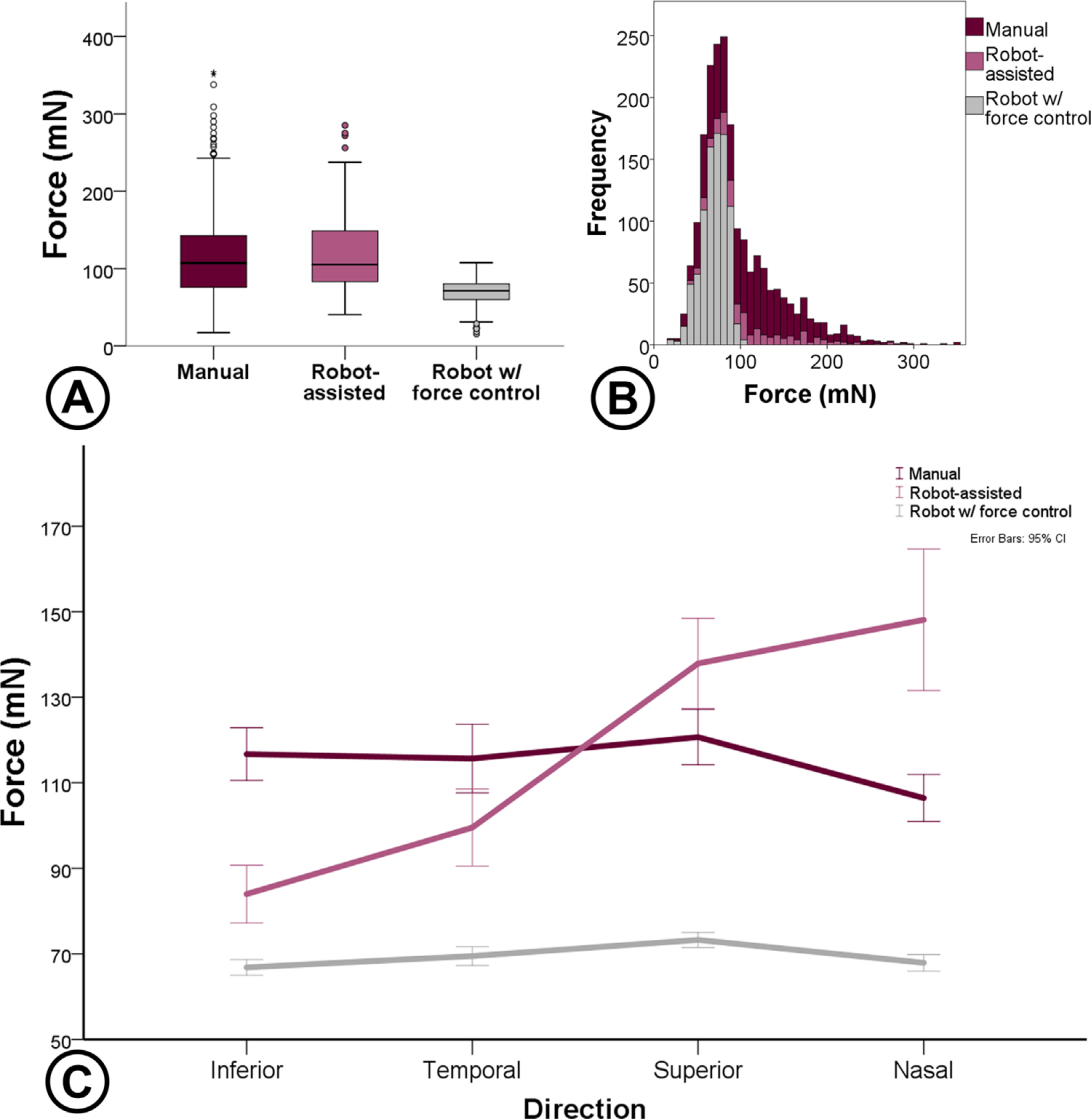
(**A**) Forces compared between control modes. (**B**) Histogram of mean forces from each attempt. (**C**) Mean forces related to sectors using different controls (manual, robot assisted, and robot with active force control). Lower forces were observed using the robot with active force control and a higher impact on force increase was observed on sectors superior and nasal.

A total of 1927 valid movements and 3,400,272 force data information were recorded, with a mean time of 8.8 seconds per movement. During free-hand tasks, mean forces were 115 ± 51 mN and using robotic assistance, mean forces were 118 ± 49, with no difference. After the implemented force control method, overall mean forces reduced to 69 ± 15, with a statistical difference compared with the other mentioned methods (*P* < 0.001). Analyzing intraocular directions, movement toward the superior sector produced higher forces (102 ± 45 mN), compared with other ones. However, the introduction of the robot impacted especially on superior and nasal sectors, as displayed in Figure 3.

**Figure 4.**
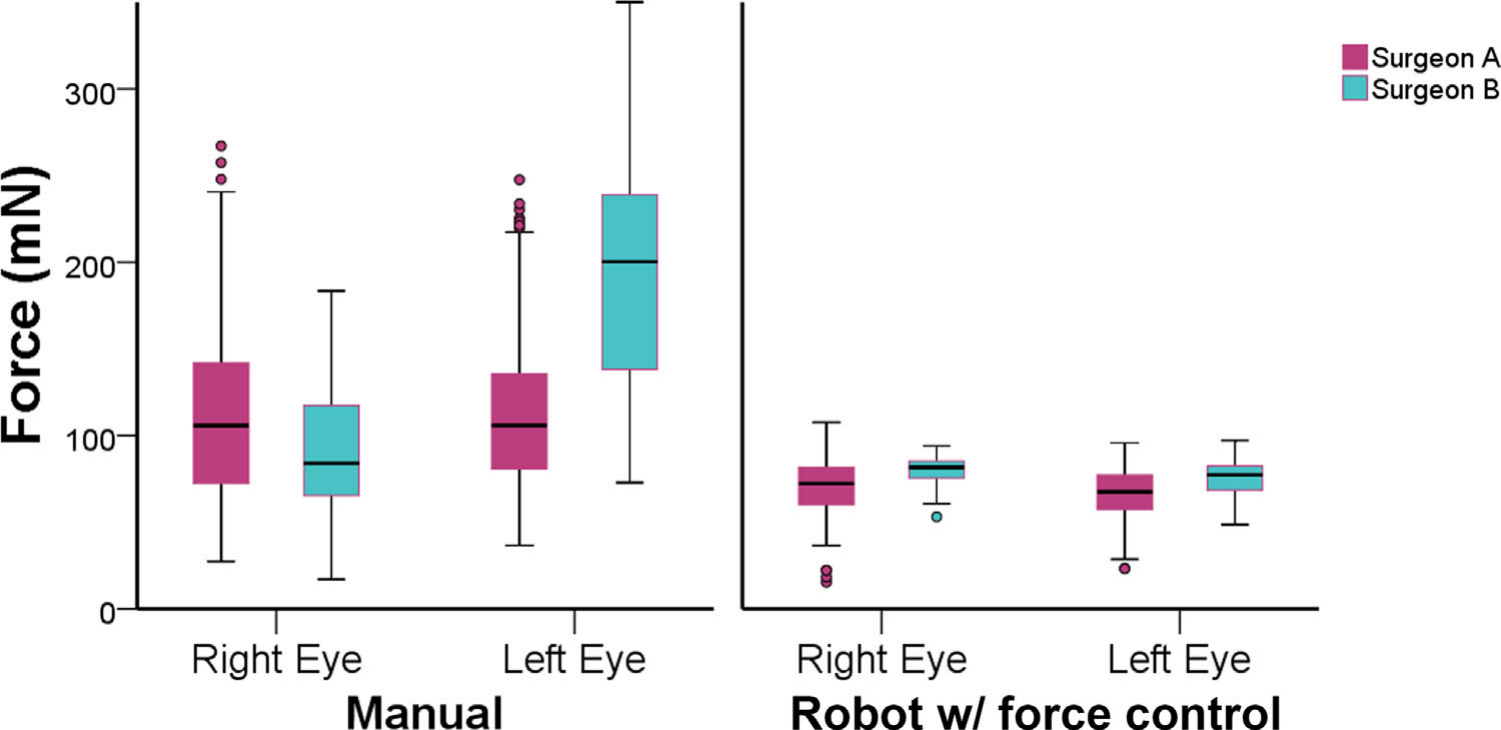
Comparison between right and left eye for different users and two methods of control. Left eye had differences in force measures using manual control.

**Figure 5.**
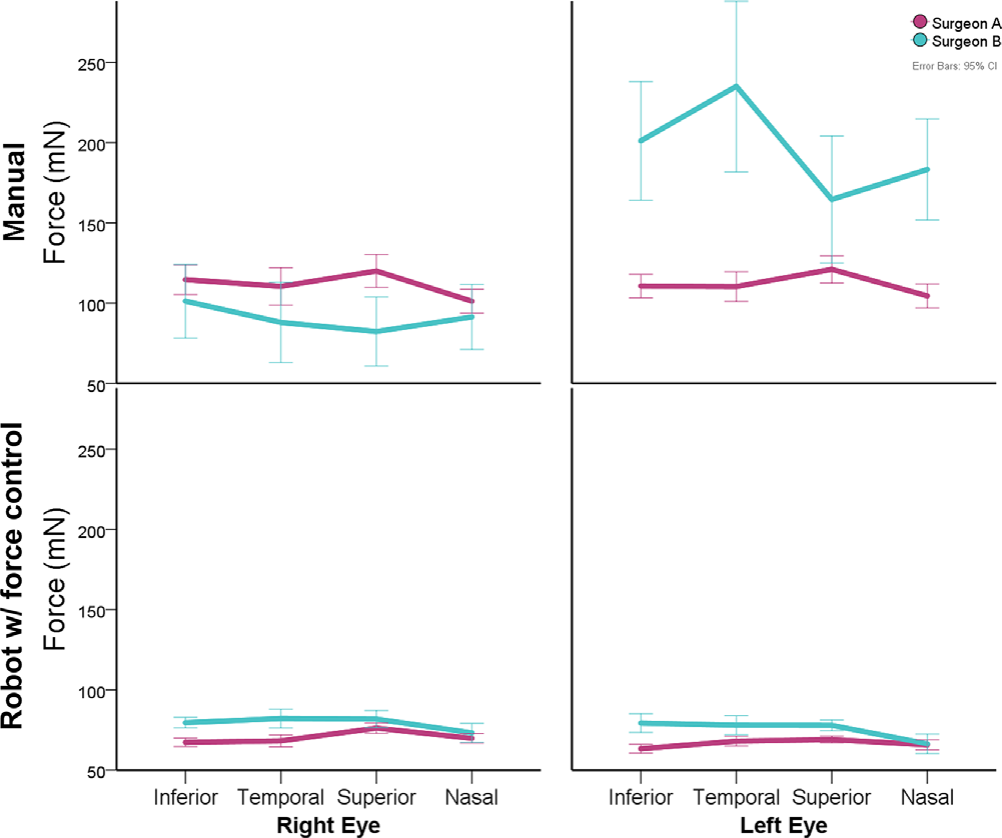
Comparison between right and left eye for different users and two methods of control. Using robotic assistance reduced differences in measures and patterns on mean forces between users.

There was no difference in overall forces between the right (91 ± 29 mN) and left eye (93 ± 33 mN), *P* = 0.255. However, a difference was observed using manual manipulations on left eye between users (surgeon A 111 ± 41 mN; surgeon B 196 ± 67 mN; *P* = 0.001), as illustrated in Figure 4. However, after implementing an active robotic force control, difference in force average between users was overall similar (see Fig. 4) and also force pattern on different directions were similar between users, despite differences in mean values (Fig. 5).

**Figure 6.**
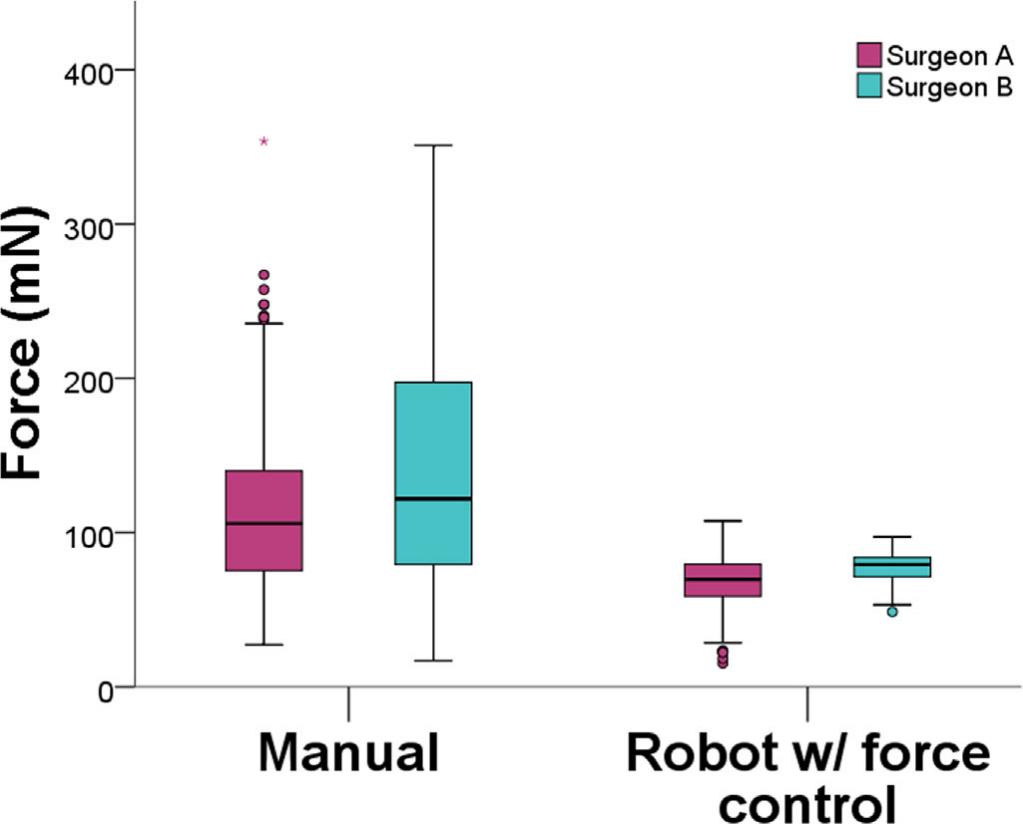
Force comparison between users and methods of control. During manual manipulations, differences were higher. Using robotic assistance, force measures were similar.

**Figure 7.**
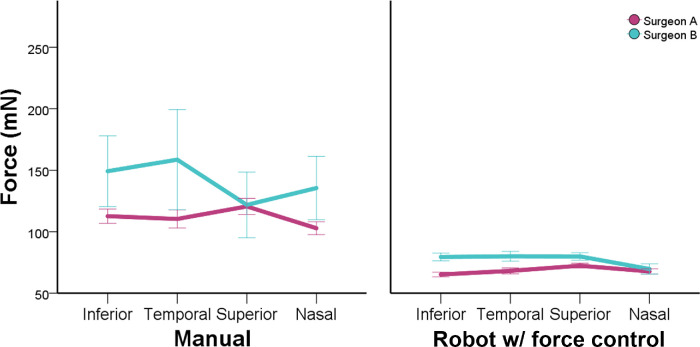
Force pattern distribution for intraocular directions comparing users and methods of control. Using robotic assistance, force pattern between users similar.

There was a difference in overall forces between surgeon A (90 ± 31) mN and surgeon B (109 ± 32 mN). This difference was verified mainly on free-hand manipulations (111 ± 38 mN vs. 140 ± 42 mN, *P* = 0.001). Although with smaller standard deviations, the use of robotic assistance with force control also have differences (68 ± 24 mN vs. 77 ± 22 mN, *P* = 0.001), as showed in Figure 6. The force pattern for different directions, however, was similar using robotic assistance, as shown in Figure 7.

## Discussion

Microsurgical interactions in vitreoretinal procedures require coordination for movement and accuracy in the order of micrometers. Robotic systems have been shown to improve access in confined spaces, but they still seek to prove their efficiency and safety in ophthalmology. Challenges in robotic assistance adhesion in general surgery are related to many factors, one of them being the incompatibility of force feedback in robots without sensors.^26^^,^^27^ Those concerns lead to surgical platforms to develop and adopt force feedback.^28^^–^^30^ In ophthalmology, robotic assistance has already been tested extensively in experimental models in some animals and even in a few humans. However, human studies did not use in their platforms any sort of force sensing and force feedback. This lack of sensing might impact in robotic manipulation and also on surgical awareness. In a field in which most forces are below human perception, force sensors might have numerous applications within instruments and might improve safety in using robotics in clinical environments.

This study mapped forces during intraocular instrument manipulation using in vivo models and compared manipulations using freehand, robot-assisted with and without force control. Results suggest that forces found using this robotic platform on previous experimental studies^31^^–^^36^ may be reduced using force control methods. We also validate benefits of a force control method using feed from sensors with a reduction of 42% of overall forces. In addition, results were similar to dry phantom experiments. This implies that studies using experimental models could be useful to test force control algorithms with less use of animals for validation. Results also could help optimize movements and instrument positioning during automated procedures in the future.

This study has some drawbacks, for example, the size of rabbit's eyes and the number of users. With regard to the animal model, we agree that a porcine model would be more similar to a human model. However, a large pig would require an increased amount of drugs and their large scale would probably impact on study sample size. Besides, extraocular muscles on rabbits are similar to humans and such a model would be enough to verify the pattern of the force map for intraocular movements. With regard to the number of users, there was indeed a restriction and certainly introduced individual bias. However, keeping the same surgeon enabled us to compare between this model and other experiments with dry phantoms and ex vivo eyes with this same surgeon.

Ultimately, one could criticize that the active force control from the robot could impact on restrictions to the user experience, and ultimately that scleral forces were never a matter of concern to surgeons, given the strength of such tissue. However, force interaction can influence the correct manipulation of the instrument with robotic assistance in clinical environments and impact on surgical awareness, especially on master-slave devices (although we cannot extend our results to those devices not tested yet). In addition, similarities of results between in vivo and dry phantoms could assist testing methods of active force control to improve user experience. Therefore, comprehending those forces might impact the correct robot manipulation and could improve surgical awareness with robotics. Besides, controlling unpredictable surgical variables might aid platform safety and enable the advertised benefits and the over-marketed hype from robotic assistance.

Nevertheless, the results of this study do not imply that the existence of a force sensor is essential for robotic assistance, however, we validate previous results and raise concerns to surgical awareness using those devices, and this study being related to this specific - cooperative robotic - platform. Different mechanisms could be equally useful for that purpose. Studies using other information sources, such as image and joint data, show benefits of numerous sources of data for kinematics awareness. Therefore, not only sensors but improvements on surgical interface, neural networks, and kinematics could impact on instrument proprioception and consequently benefit procedure safety. All marketing benefits from extreme accuracy and stability are indeed insightful for the utility of robotic assistance. However, if the robot use does not prove to be safe, then improving accuracy for a clinical use might be pointless.

## Conclusions

Robotic systems can be useful in micrometric environments that require accuracy and security, but this introduction raises concerns with safety. This study validates previous findings in experimental models, and shows increased forces with the introduction of the robotic assistance. Additionally, we also described benefits of robotic assistance when a force control method was applied using feed from a force sensing instrument to the robot. Numerous applications might exist with the potential features of robotic assistance in ophthalmology. However, improving extreme accuracy and other robotic features make the most sense when concerns are addressed and safety is established.
